# Hexanuclear Cu_3_O–3Cu triazole-based units as novel core motifs for high nuclearity copper(ii) frameworks†

**DOI:** 10.1039/c9ra05922a

**Published:** 2019-09-17

**Authors:** Sacramento Ferrer, Javier Hernández-Gil, Francisco Javier Valverde-Muñoz, Francisco Lloret, Alfonso Castiñeiras

**Affiliations:** Departament de Química Inorgànica, Universitat de València Av. Vicent Andrés Estellés, s/n, 46100 Burjassot Valencia Spain Sacramento.Ferrer@uv.es; Institut de Ciència Molecular (ICMol), Universitat de València C/Catedrático José Beltrán Martínez, 2, 46980 Paterna Valencia Spain; Departamento de Química Inorgánica, Facultad de Farmacia, Universidad de Santiago de Compostela Praza do Seminario de Estudos Galegos, s/n 15782 Santiago de Compostela Spain

## Abstract

The asymmetric 3,5-disubstituted 1,2,4-triazole ligand H_2_V (5-amino-3-picolinamido-1,2,4-triazole) by reaction with an excess of Cu(ii) perchlorate (Cu : H_2_V being 12 : 1) has produced a novel hexanuclear {Cu_6_(μ_3_-O/H)(HV/V)_3_} fragment, with one triangular Cu_3_(μ_3_-O/H) group connected to three peripheral single Cu(ii) ions through a *cis*–*cis*–*trans* bridging mode of the ligand, which is the building block of the three structures described here: one hexanuclear, [Cu_6_(μ_3_-O)(HV)_3_(ClO_4_)_7_(H_2_O)_9_]·8H_2_O (1), one dodecanuclear, [Cu_12_(μ_3_-O)_2_(V)_6_(ClO_4_)_5_(H_2_O)_18_](ClO_4_)_3_·6H_2_O (2), and one tetradecanuclear 1D-polymer, {[Cu_14_(μ_3_-OH)_2_(V)_6_(HV)(ClO_4_)_11_(H_2_O)_20_](ClO_4_)_2_·14H_2_O}_*n*_ (3), the last two containing hexanuclear subunits linked by perchlorato bridges. The Cu–Cu av. intra-triangle distance is 3.352(2) Å and the Cu(central)–Cu(bridged external) av. distance is 5.338(3) Å. The magnetic properties of the hexanuclear “Cu_3_O–3Cu” cluster have been studied, resulting as best fit parameters: *g* = 2.18(1), *J*(intra-triangle) = −247.0(1) cm^−1^ and *j*(central Cu^II^ – external Cu^II^) = −5.15(2) cm^−1^.

## Abstract 2

The novel hexanuclear {Cu_6_(μ_3_-O/H)(HV/V)_3_} fragment is the building block of the hexanuclear, the dodecanuclear and the tetradecanuclear-1D-polymeric structures described here.

## Graphical abstract

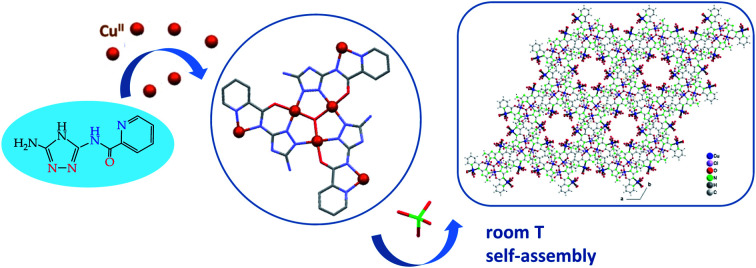

## Introduction

Polynuclear Cu(ii) compounds are of interest in biology and in magnetochemistry.^1^ The structural and electronic factors that govern exchange coupling have been well stablished in small di-, tri- and tetrametallic clusters.^2^ More recently, the pursuit of single molecule magnets has focused the attention on ever larger clusters with higher multiplicity ground states.^3^ In addition, the last few years have witnessed an impressive growth of the literature on 1D, 2D and 3D coordination polymers (CPs) with the aggregates of Cu(ii) compounds emerging as a type of subunit whose (self)assemblies may form supramolecular structures.^4^

The rich bridging chemistry of the 1,2,4-triazole ligands towards first-row transition metals has produced multiple nuclearities and topologies.^5,6^ We have previously reported a series of 3-substituted-1,2,4-triazole derivatives which are able to generate trinuclear copper-complexes containing the [Cu_3_(μ_3_-OH)(trz)_3_]^*n*+^ core.^7,8^ This specific class of tricopper clusters (with trigonal symmetry) are receiving great attention for two reasons: (1) because of their magnetic singularity, which involves geometric spin frustration effects, antisymmetric exchange coupling and unusual electron paramagnetic resonance (EPR) response,^7–9^ features which make them relevant as magnetic models to understand the mechanism of action of multicopper enzymatic systems;^10^ and (2) as subunits (secondary building units, SBU) of metal–organic frameworks (MOFs, also termed porous coordination polymers, PCPs) which combine magnetism and porosity.^11,12^

Literature on triangular Cu_3_O/OH arrays has basically been focused on oximato (N,O), pyrazole (N,N), 1,2,4-triazole (N,N) and more recently 1,2,3-triazole (N,N) and tetrazole (N,N) bridging groups.^13^ The assembly of triangular units in larger aggregates has been achieved (a) by dimerization of the trimeric units through H-bonds between μ_3_-O/μ_3_-OH centered triangles,^14,15*a*^ (b) through bridging counteranions,^15*b*,*c*^ (c) by means of bi-topic amines^15*d*,*e*^ or carboxylate bridges/bis-carboxylato linkers,^15*f*,*g*^ and (d) with the use of carboxylate-functionalized N,N ligands (construction of MOFs).^16–19^

In oximate systems, some hexanuclear systems^20^ and a few catena^21^ have been reported to date. In contrast, for the pyrazole family there are many examples of hexacopper complexes and of chains of Cu_3_O fragments grown with chlorido, sulfato, perchlorato, thiocyanato, carboxylato or bipyridine bridges.^15^

As for the 1,2,4-triazol systems, Cu_3_O/OH assembles are more scarce in number but more diverse than the pyrazole ones,^13^ and the corresponding works could be classified in three groups. (A) The most prolific one comprises a series of fascinating 2D and 3D structures mostly obtained from solvothermal synthesis by self-association of bifunctional triazole-carboxylate/carboxylic ligands,^16–18^ triazole-isophthalate ligands,^19^ or ternary triazolate-sulfoisophthalate systems.^11,12^ (B) A second group includes the studies which combine triazole ligands with polyoxometalates to render POM-based trinuclear clusters.^22–24^ (C) The third class contains a few examples of polymers made up with simple triazole ligands in which the triangular Cu_3_O motifs are bridged by ditopic anions such as sulfonate^25^ or nitrate,^14^ as well as one hexanuclear structure with an unusual μ_6_-Cl^−^ anion.^26^ There are also two special cases with a Cu(ii) center linking two Cu_3_O units.^7*b*,25^ Finally, it has been described one related tetrazole hexanuclear copper complex constructed with sulfato bridges.^27^

In this report we introduce the small polydentate 1,2,4-triazole ligand which not only affords triangular Cu_3_O cores but also chelates in *trans* one external Cu(ii) center to give an unprecedented Cu_3_O–3Cu (Cu_3_ + Cu + Cu + Cu) hexanuclear compound with formula [Cu_6_(μ_3_-O)(HV)_3_(ClO_4_)_7_(H_2_O)_9_]·8H_2_O (1). Further on, the linkage of two hexanuclear motifs through μ_2_/μ_3_-perchlorate anions has yielded one dodecanuclear discrete compound and one tetradecanuclear 1D-coordination polymer formulated as [Cu_12_(μ_3_-O)_2_(V)_6_(ClO_4_)_5_(H_2_O)_18_](ClO_4_)_3_·6H_2_O (2) and {[Cu_14_(μ_3_-OH)_2_(V)_6_(HV)(ClO_4_)_11_(H_2_O)_20_](ClO_4_)_2_·14H_2_O}_*n*_ (3), respectively. The three structures and the magnetic properties of this new type of hexanuclear cluster are presented.

## Experimental

### Synthesis of 1, 2 and 3

Synthesis of the ligand H_2_V was performed as previously reported.^28^

A methanolic suspension (20 mL) of H_2_V (0.25 mmol, 0.052 g) was stirred for 20 min and then heated with stirring at 50 °C for 10 min (ligand only partially solved). At that point a methanolic solution (5 mL) of Cu(ClO_4_)_2_·6H_2_O (3 mmol, 1.111 g) was added dropwise and the mixture stirred for 1 h. The resulting green suspension was filtered off. Additional methanol (2.5 mL) was added to the filtered solution, which was deposited on a crystallizing dish (initial reactants ratio is H_2_V : Cu(ii) = 1 : 12). Cubic-like green crystals of 1 suitable for X-ray analysis were obtained after *ca.* two months. Yield: *ca.* 60%. IR (ATR, cm^−1^): 3459(m), 3363(m), 1660(s, sharp), 1617(m), 1588(s, sharp), 1532(s, sharp), 1484(w), 1434(w), 1394(s, sharp), 1307(w), 1266–1241(w), 1054(vs), 985(w, shoulder), 929(w). Anal. calc. for C_24_H_55_Cl_7_Cu_6_N_18_O_49_ (1) (2009.25): C, 14.35; H, 2.76; N, 12.55, Cl, 12.35. Found: C, 14.00; H, 2.60; N, 11.96; Cl, 11.98. The synthesis was reproduced several times. Some of the crystallizations (the most concentrated, approx. 1 out of 5) rendered crystals of different shapes. The X-ray study revealed that the most abundant, the cubic-like ones, correspond to compound 1 (approx. 55% yield). A few of them, hexagonal-prismatic shaped, correspond to compound 2 (approx. 4% yield). Finally, a third type, big long-prismatic shaped, corresponds to compound 3 (approx. 1% yield).

### Crystal structure determination

Crystal data, data collection and structure refinement details for 1, 2 and 3 are summarized in Table 1. Diffraction data were obtained at 100(1) K using a Bruker SMART CCD 1000 (1 and 2) or a Bruker X8 Kappa APEXII (3) diffractometer from crystals mounted on glass fibers. Data were corrected for Lorentz and polarization effects and for absorption following multi-scan type.^29^ The structures were solved by direct methods and subsequent difference Fourier maps^30^ and refined on *F*^2^ by a full-matrix least-squares procedure using anisotropic displacement parameters.^30^ Hydrogen atoms attached to carbon and nitrogen atoms were placed in geometrically idealized positions and were refined with isotropic displacement parameters constrained to 1.2/1.5 Ueq of the carrier atoms. Molecular graphics were generated with DIAMOND.^31^ For each structure peculiarities of the refinement are indicated in ESI (S1–S3).†

**Table 1:** Crystal and structure refinement data for 1–3

	1	2	3
Chem. formula	C_24_H_55_Cl_7_Cu_6_N_18_O_49_	C_48_H_84_Cl_8_Cu_12_N_36_O_64_	C_56_H_113_Cl_13_Cu_14_N_42_O_95_
*M*_r_	2009.25	3235.59	4245.29
Cryst. size (mm)	0.260 × 0.230 × 0.220	0.240 × 0.150 × 0.100	0.320 × 0.260 × 0.120
Cryst. syst.	Trigonal	Hexagonal	Monoclinic
Space group	*R*3̄:H	*P*6_3_/*m*	*C*2/*c*
*a* (Å)	20.496(3)	20.811(3)	35.294(3)
*b* (Å)	20.496(3)	20.811(3)	20.8571(17)
*c* (Å)	33.675(6)	22.620(3)	23.370(3)
*α* (°)	90	90	90
*β* (°)	90	90	98.149(5)
*γ* (°)	120	120	90
*V* (Å^3^)	12 251(4)	8484(3)	17 030(3)
*Z*, *D*_c_ (g cm^−3^)	6, 1.634	2, 1.267	4, 1.656
*h*/*k*/*l*	−25/12, 0/25, 0/42	−18/0, 0/21, 0/23	−39/38, 0/23, 0/25
*F*(000)	6060	3240	8520
Absorption coeff. (mm^−1^)	1.865	1.673	2.019
No. of collected/unique rflns	33712/5630	47107/3580	12 143/12 143
*R*_int_	0.0498	0.0542	0.0516
No. of data/restraints/params	5630/67/318	3580/13/301	12 143/148/777
*R*_1_/*wR*_2_ (*I* > 2σ(*I*))	0.1021/0.2846	0.0798/0.2308	0.1198/0.2663
*R*_1_/*wR*_2_ (all data)	0.1521/0.3377	0.1125/0.2547	0.1768/0.2873
Max, min transmission	1.0000, 0.7645	1.0000, 0.7555	1.0000, 0.8926
GOF on *F*^2^	1.049	1.041	1.096
Δ*ρ*_max_, Δ*ρ*_min_ (e Å^−3^)	1.939, −1.352	1.407, −0.503	3.268, −1.283
CCDC number	1935979	1935980	1935981

### Magnetic measurements

Magnetic susceptibility measurements on polycrystalline samples were carried out with a Superconducting Quantum Interference Design (SQUID) magnetometer in the temperature range of 1.9–300 K under magnetic fields of 250–5000 Gauss. Diamagnetic corrections of the constituent atoms were estimated from Pascal's constants. Experimental susceptibilities were also corrected for the temperature independent paramagnetism, *χ*_TIP_ = 60 × 10^−6^ cm^3^ mol^−1^ per copper(ii), and for the magnetization of the sample holder. X-Band EPR spectra of polycrystalline samples were recorded at different temperatures with a Bruker ER 200 spectrometer equipped with a helium continuous-flow cryostat.

## Results

### The ligand

**Scheme 1:**
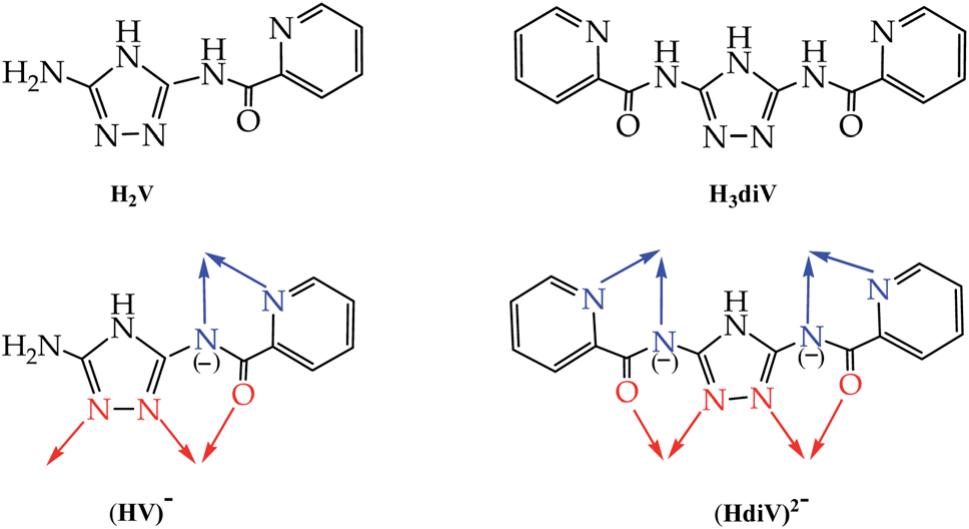
Comparing ligands H_2_V and H_3_diV: bridging mode of the mono-deprotonated (HV)^−^ (or the di-deprotonated V^2−^) (this work) and the di-deprotonated (HdiV)^2−^ ligands.^32^

**Scheme 2:**
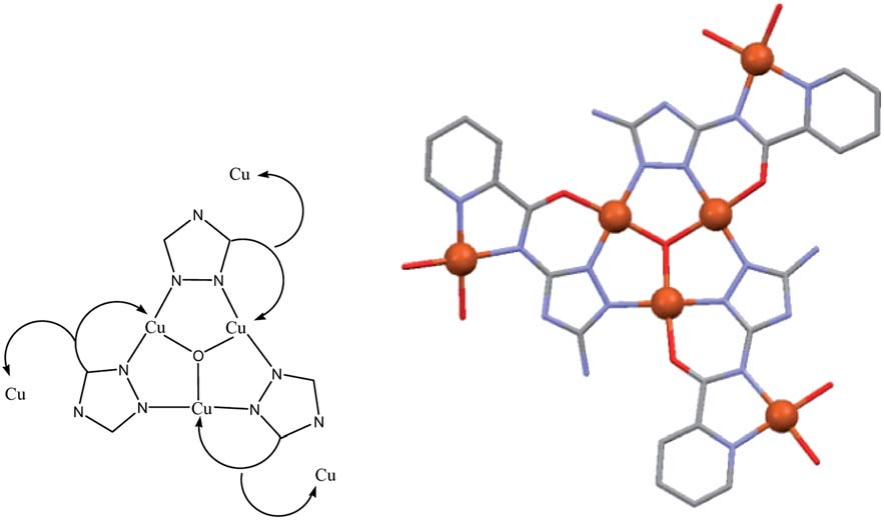
The quiral “3 + 1 + 1 + 1” hexanuclear copper(ii) core-motif (right: Cu atoms in red ball). The average (comp. 1 + comp. 2 + comp. 3) distances are: Cu–Cu(intra-triangle) = 3.352(2) Å, and Cu(central)–Cu(closest external) = 5.338(3) Å.

The ligand of the present study, H_2_V [5-amino-3(pyridine-2-yl-acetamido)-1,2,4-triazole=5-amino-3-picolinamido-1,2,4-triazole], was obtained from mono-acetylation of the guanazole with picolinic acid.^28^ In a previous work we reported on a similar ligand, the H_3_diV [3,5-bis(picolinamido)-1,2,4-triazole], synthetized by di-acetylation of the guanazole (Scheme 1). From the previous H_3_diV ligand and with an excess of copper(ii) perchlorate a hexanuclear cationic complex of formula [Cu_6_(HdiV)_2_(ClO_4_)_6_(H_2_O)_14_]^2+^ was formed, which could be described as a cluster of the “1 + 1 + 2 + 1 + 1” type.^32^ The ligand H_2_V, a 1,2,4-triazole ligand bearing only one chelating arm, studied here as such for the first time, in the presence of Cu(ii) forms the Cu_3_N_6_ nine-membered ring of the Cu_3_O(H) triangular family.^7,8^ The feature which makes H_2_V unique is the bis-chelating nature of the triazole substituent which links in *trans* one additional atom centre, thus resulting in the “3 + 1 + 1 + 1” hexanuclear copper(ii) core-motif (Scheme 2). From this building block three different structures have been formed which we present here: one hexanuclear Cu_6_ (1), one di-hexanuclear (2 × Cu_6_=Cu_12_) (2), and one 1D-polymer made of dimers linked by pairs of hexamers (Cu2–Cu6–Cu6–Cu2′, Cu14) (3).

### Description of structures

#### Crystal structure of [Cu_6_(μ_3_-O)(HV)_3_(ClO_4_)_7_(H_2_O)_9_]·8H_2_O (1)

**Fig. 1:**
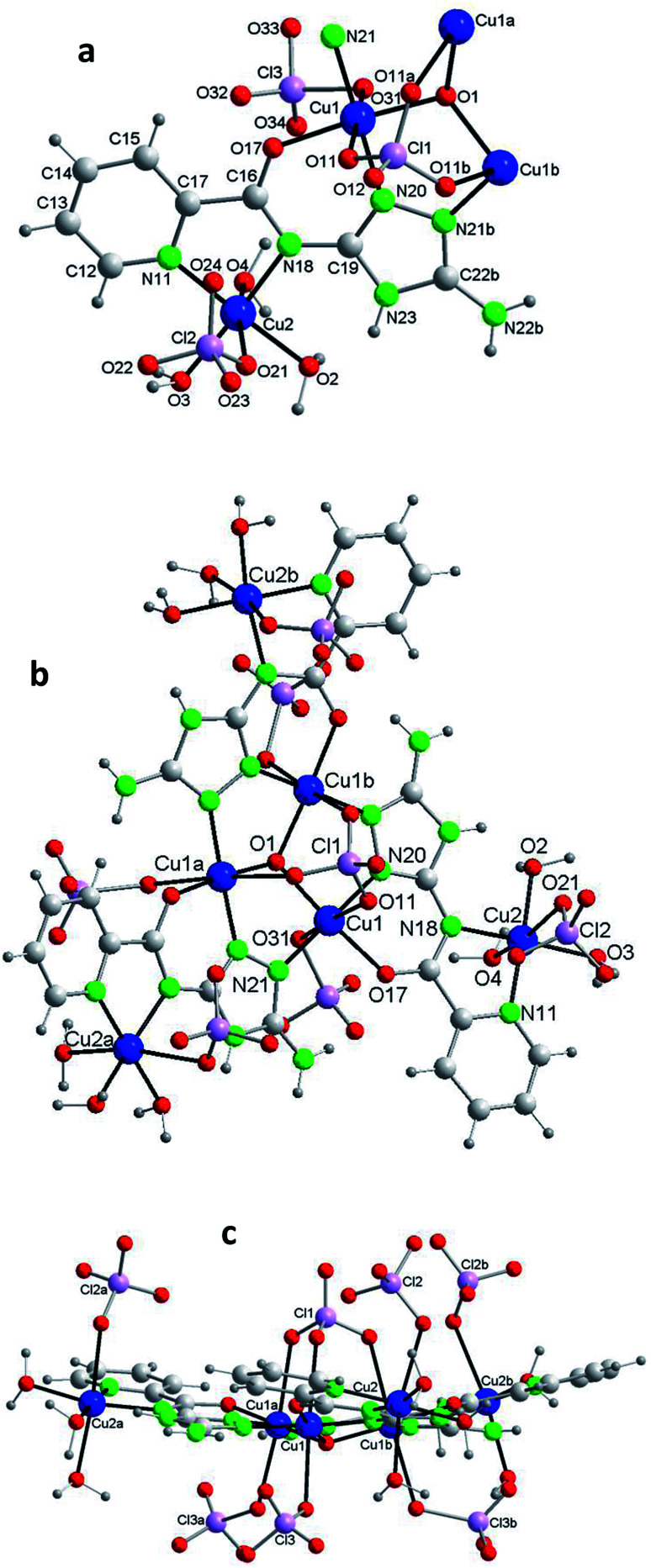
Compound 1. (a) The asymmetric unit with the labelling [symmetry codes: (a) −*y* + 1, *x* − *y* + 1, *z*; (b) −*x* + *y*, −*x* + 1, *z*]. (b) The hexanuclear compound. (c) The hexanuclear compound (view perpendicular to the *c* axis). (Water molecules of crystallization omitted for clarity.)

Compound 1 crystallizes in the trigonal system (Table 1). The unit cell contains 6 neutral [Cu_6_(μ_3_-O)(HV)_3_(ClO_4_)_7_(H_2_O)_9_] complexes and 8 water molecules of crystallization (Fig. 1). Table 2 lists selected distances and angles (full list in S4†). The ligand H_2_V is deprotonated at the N acetamido group and retains the H atom of the 1,2,4-triazole ring (the H atom is on N4 (N23); this tautomer differs from that present in the reported crystal structure of the ligand).^28^ The hexanuclear compound includes two type of copper centers, Cu1 and Cu2. There are three Cu(ii) ions symmetrically related (Cu1, Cu1a, Cu1b) in a triangular arrangement hold peripherally by N–N triazole bridges and in the center by a μ_3_-oxo bridge. The coordination environment of these Cu(ii) ions is a distorted octahedron, with two N-triazole atoms, one O-carbonyl atom and the central oxo anion in equatorial positions, and two O-perchlorate atoms in the apical ones. There are four coordinating perchlorate anions in total since one of them (Cl1) is tridentate and coordinates simultaneously the 3 copper(ii) ions of the {Cu_3_O} core, at a Cu(1)–O(11) distance of 2.63(2) Å. For oximato (N,O), pyrazole (N,N) and 1,2,4-triazole (N,N) systems, it has been described that Td anions (such as sulfate, perchlorate, *etc.*) often act as tridentate ligands and block three axial coordination sites on the same side of the triangle.^15*b*,*c*,20*a*,24,25^

**Table 2:** Selected bond lengths (Å) and angles (°) for 1–3^a^^,^^b^

	1	2	3
Cu(1)–N(20)	1.909(6)	1.903(6)	1.912(10), 1.910(10), 1.895(10)
Cu(1)–N(21)	1.957(6)	1.947(6)	1.945(10), 1.935(10), 1.955(11)
Cu(1)–O(17)	1.969(6)	1.964(5)	1.968(9), 1.968(9), 1.972(9)
Cu(1)–O(1)	1.995(3)	2.009(3)	1.986(8), 1.994(9), 2.012(8)
Cu(1)–O(31)/*O(21)*/**O(w)**	2.501(11)	2.417(7)	2.403(13), 2.385(11), 2.387(17)
Cu(1)–O(11)	2.634(14)	2.506(8)	2.450(12), 2.852(19), 2.546(15)
Cu(1)#1–O(1)–Cu(1)	113.8(3)	113.6(2)	114.8(4), 113.1(4), 114.1(4)
Cu(1)–Cu(1)#1	3.344(2)	3.362(2)	3.335(2), 3.353(2)
O(1)–O(1)′	5.208(9)	5.648(18)	6.816(9)
Cu(2)–O(3)	1.972(8)	1.933(18)AA/2.014(16)BB	1.988(12), 1.965(12), 1.995(11)
Cu(2)–N(11)	1.968(8)	2.009(9)A/1.971(8)B	1.974(12), 1.983(11), 1.960(12)
Cu(2)–O(2)	1.994(8)	2.005(18)AA/2.005(19)BB	1.989(12), 2.003(11), 1.94(15)
Cu(2)–N(18)	2.008(6)	2.011(8)A/2.010(8)B	2.009(11), 2.003(10), 2.007(12)
Cu(2)–O(21)/**O(x1)/O(w)**	2.470(11)		2.265(18), 2.422(15), 2.258(19)
Cu(2)–O(4)/**O(y1)**	2.69(3)	2.21(4)AA/2.39(6)BB	2.469(13), 2.456(17), 2.423(14)
Cu(1)–Cu(2)	5.345(2)	5.256(3)A/5.365(3)B	5.361(2), 5.367(2), 5.352(3)
*Cu(1)–Cu(1)&3*		6.687(2)	
*Cu(1)–Cu(1)&4*		7.485(2)	
**Cu(7)–O(77)/Cu(8)–O(103)**			1.76(3)/1.88(3)
**Cu(7)–N(80)/Cu(8)–N(71)**			1.85(3)/1.964(16)
**Cu(7)–O(101)/Cu(8)–N(78)**			1.94(5)/1.95(3)
**Cu(7)–O(102)/Cu(8)–O(104)**			2.11(6)/2.00(3)
**Cu(7)–O(52)/Cu(8)–O(14)**			2.67(2)/2.498(18)
**Cu(7)–Cu(8)**			5.272(9)

a For 1: symmetry transformations used to generate equivalent atoms: #1 −*y* + 1, *x* − *y* + 1, *z*, #2 −*x* + *y*, −*x* + 1, *z*. For 2: symmetry transformations used to generate equivalent atoms: &3 *x*, *y*, −*z* + 1/2, &4 −*x* + *y* + 1, −*x* + 1, −*z* + 1/2.

b Labels referred to compound 1 (Fig. 1a and b); for compounds 2 and 3 the distances listed correspond to the equivalent bonds. In italic, labels specific of compound 2; in bold labels specific of compound 3. For complete tables of distances and angles see S4–S6.

Each of the 3 (HV)^−^ ligands bridges two Cu(ii) ions of the Cu_3_O group while chelating one of them (bite angle of 86.5(2)°). Besides, each ligand chelates in *trans* another Cu(ii) center, Cu2 (bite angle of 82.5(2)°) (Scheme 2, Fig. 1a and b). So, in whole, there are 3 external single Cu(ii) atoms (Cu2, Cu2a, Cu2b). This second type of copper center exhibits a very distorted tetragonally octahedral stereochemistry, in which one N-amido, one N-pyridine and two O-water atoms occupy the basal sites and one O-water molecule and one O-perchlorate atom (at 2.47(2) Å) the axial ones.

In the triangular array, the Cu(1)–O(1) distances are of 1.995(3)Å and the Cu(1)–O(1)–Cu(1a) angles of 113.8(2)°. The central oxygen lies 0.51(1) Å below the plane defined by the three copper atoms. In spite of this deviation of the Cu_3_O moiety from planarity, the lack of H-bonding together with the charge balance (and the symmetry of the molecule) lead us to exclude the existence of the central oxygen as OH species (as found in related triangular triazole compounds)^7,8^ instead of O. The intra-triangle Cu1–Cu1 distance, 3.344(2) Å, is in the range observed for related systems.^7*a*,8^ The Cu1–Cu2 distance is 5.345(2) Å.

A view perpendicular to the *c* axis shows the {Cu_6_(μ_3_-O)(HV)_3_} fragment defining a rough plane (Fig. 1c). The hexanuclear unit is chiral although, since in the network there are inversion centers, the bulk solid is racemic (*i.e.* the compound is achiral). Above the plane there are 4 perchlorate anions, three monodentate (towards each of the external Cu(ii) centers) and the mentioned tridentate (which bridges the three central Cu(ii) atoms); below the plane another 3 monodentate perchlorato ligands complete the octahedral coordination of the three Cu_3_O copper atoms. The internal perchlorates together with the axially coordinated water molecules connect one {Cu_6_(μ_3_-O)(HV)_3_} unit with an upside-down second {Cu_6_(μ_3_-O)(HV)_3_} unit, of opposite quirality, through H-bonds (Fig. S7†). The O1⋯O1′ distance is 5.208(9) Å. In turn, the external perchlorate anions bind each pair of hexamers with another pair *via* additional H-bonds thus giving chains of pairs of hexanuclear compounds (Fig. S8†).

#### Crystal structure of [Cu_12_(μ_3_-O)_2_(V)_6_(ClO_4_)_5_(H_2_O)_18_](ClO_4_)_3_·6H_2_O (2)

**Fig. 2:**
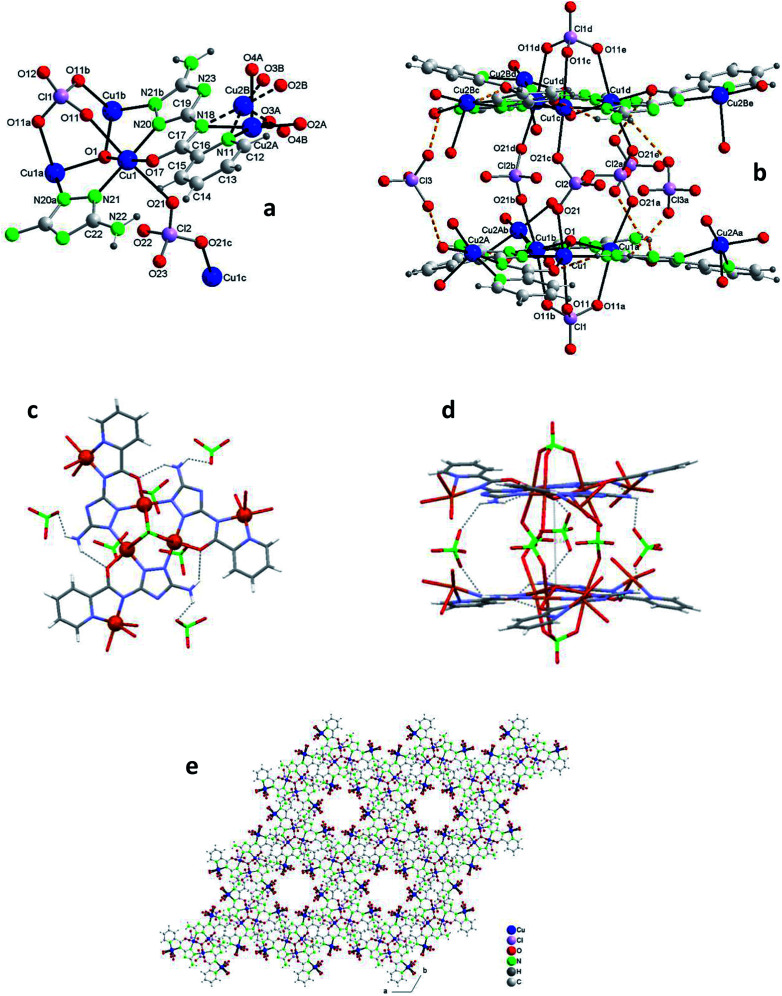
Compound 2. (a) The asymmetric unit with the labelling and the two alternative orientations for the O(2)–Cu(2)–O(3) fragment [symmetry codes: (a) −*y* + 1, *x* − *y*, *z*; (b) −*x* + *y* + 1, −*x* + 1, *z* and (c) *x*, *y*, −*z* + 1/2]. (b) The dodecanuclear compound (along *a*) (hydrogen bonds shown as orange dashed lines) [symmetry codes: (a) −*y* + 1, *x* − *y*, *z*; (b) −*x* + *y* + 1, −*x* + 1, *z*; (c) *x*, *y*, −*z* + 1/2 (d) −*x* + *y* + 1, −*x*+1, −*z* + 1/2 and (e) −*y* + 1, *x* − *y*, −*z* + 1/2]. (c) Schematic view (projected on plane *ab*) (Cu atoms in red ball; shown selected H-bonds). (d) Schematic view (along *c*) (shown selected H-bonds). (e) Packing (projected on plane *ab*) showing the channels. (Water molecules of crystallization omitted for clarity.)

Compound 2 crystallizes in the hexagonal system (Table 1). The unit cell contains 2 cationic [Cu_12_(μ_3_-O)_2_(V)_6_(ClO_4_)_5_(H_2_O)_12_]^3+^ complexes, perchlorate anions and water molecules of crystallization (Fig. 2). Table 2 lists selected distances and angles (full list in S5†). In this case the ligand H_2_V is deprotonated both at the N acetamido group and at the triazolato ring (so it is present as V^2−^).

The structure of 2, as that of 1, is based on the hexanuclear {Cu_6_(μ_3_-O)(L)_3_} block, the main difference between both being that in 2, two hexanuclear units related by one reflection plane (Fig. 2c and S7†) are linked by 3 perchlorate bidentate anions to give a dodenuclear Cu(ii) compound (Fig. 2b and d). The O(1)⋯O(1)′ distance in 2, of 5.648(18) Å, is somewhat longer than the equivalent O(1)⋯O(1)′ distance in paired units of 1 (5.208(9) Å). The deviation of O(1) from the Cu_3_ plane is similar (0.52(1) Å). The schematic view of Fig. 2d shows the position of the 8 ClO_4_^−^ groups: 2 tridentate external (on a 6-fold axis) and 6 interleaved, 3 of which are bidentate and connect central Cu_3_–O atoms from two opposite triangular units, and the other 3 being non-coordinating but contributing to stabilize the dodenuclear species through H-bonds. The 6 enclosed perchlorate anions are placed on a mirror plane which contains the Cl atom and two out of the four O atoms of each perchlorate.

A second difference with 1 stands on the lack of any perchlorate on the apical coordination positions of the Cu2 peripheral centers. To be noticed the disorder on these Cu2 atoms (Fig. 2a and S9†). The fragment O(2)–Cu(2)–O(3) of the metal coordination sphere has been assumed to occupy two alternative orientations inclined at an angle of 37(1)° and given an occupancy of 0.5 for each position. Fig. 2b–d display only one out of the two positions for clarity purposes. While the geometry of the central Cu1 atoms is distorted octahedral, Cu(N_2_O_2_ + O_2_) (like in 1), that of the outer Cu2 centers is Cu(N_2_O_2_ + O) penta-coordinated. Table 2 compares selected bond angles and distances in the structure of 1 and 2.

A last remarkable feature of the structure of 2 refers to the packing. A view projected on plane *ab* reveals the existence of wide channels in the network (a 18.631(9) Å void between opposite N4-triazole atoms) (Fig. 2e).

#### Crystal structure of {[Cu_14_(μ_3_-OH)_2_(V)_6_(HV)(ClO_4_)_11_(H_2_O)_20_](ClO_4_)_2_·14H_2_O}_*n*_ (3)

**Fig. 3:**
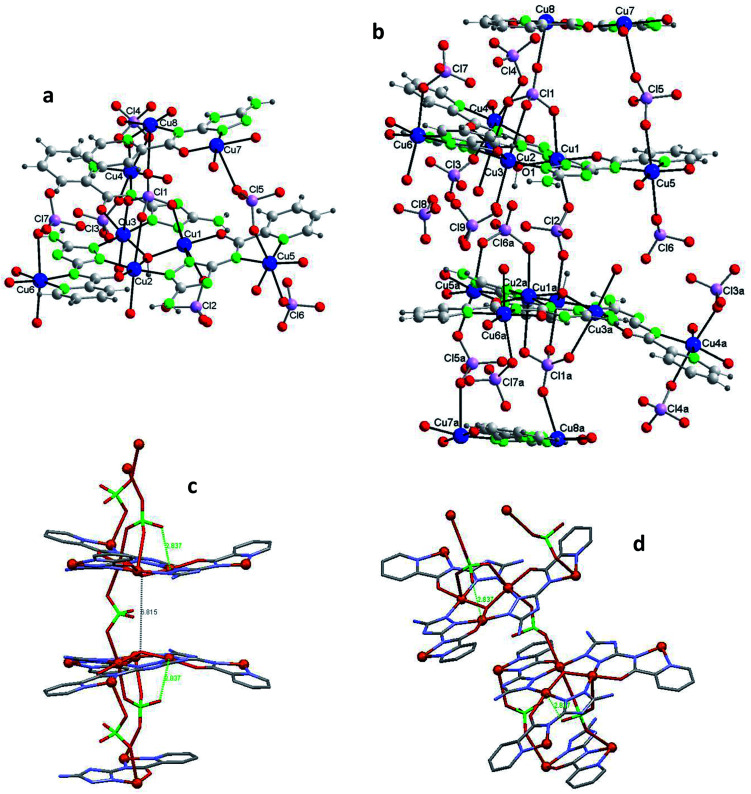
Compound 3. (a) The asymmetric unit with the labelling (perchlorate anions and water molecules of crystallization omitted for clarity). (b) The tetradecanuclear compound (along *a*) (14Cu + 2Cu′ are depicted to show the building of the chain [symmetry code: (a) −*x*, *y*, −*z* + 1/2]). (c) Schematic view (along *c*) (Cu atoms in red ball; distances only approx.). (d) Schematic view (approx. along *b*; distances only approx.). (Water molecules of crystallization omitted for clarity.)

Compound 3 crystallizes in the monoclinic system (Table 1). The intricate structure, a 1D MOF, consists of pairs of hexanuclear units, analogous to those of 1 and 2, linked through polydentate perchlorate anions to dinuclear units formed by two Cu(ii) centers *trans*-bridged by one HV^−^ ligand, which yield as a whole tetradecanuclear “⋯Cu_2_–(Cu_3_O–3Cu)–(Cu_3_O–3Cu)–Cu_2_′⋯” copper(ii) assembles. The unit cell contains 4 cationic [Cu_14_(μ_3_-O)_2_(V)_6_(HV)(ClO_4_)_11_(H_2_O)_20_]^2+^ species, perchlorate anions and guest water molecules (Fig. 3 and S10†). Table 2 lists selected distances and angles (full list in S6†). In this case the ligand H_2_V acts as V^2−^ in the hexanuclear units and as (HV)^−^ in the dinuclear ones.

Fig. 3b shows one of those 14Cu species. Fig. 3c and d are alleviated pictures in which water molecules (coordinating/crystallization) and non-coordinating/monodentate perchlorate anions are not displayed to facilitate visualization of the 14Cu centers and the different bridges that connect them (note: Fig. 3b–d contain 14 + 2 Cu).

**Fig. 4:**
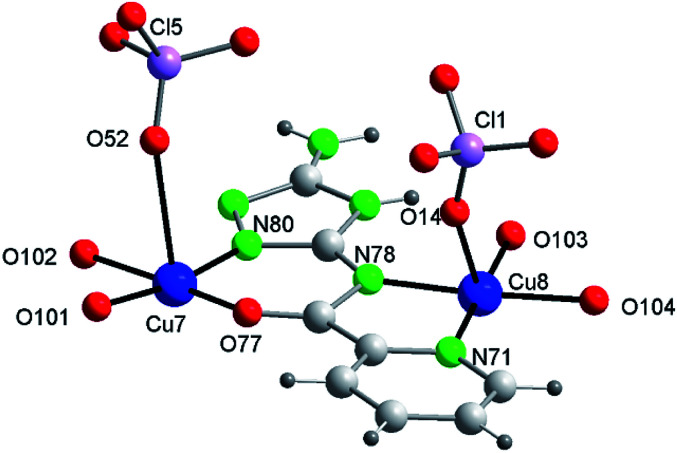
The dinuclear [Cu_2_(HV)(ClO_4_)_2_(H_2_O)_4_] fragment of compound 3 with the labelling.

The 14Cu aggregate contains 8 different (symmetry independent) Cu(ii) centers, 9 different (symmetry independent) perchlorate anions and 7 (6V^2−^ + 1(HV)^−^) ligands. Cu1, Cu2, Cu3, Cu4, Cu5 and Cu6, together with 3 ligands and one μ_3_-hidroxo ligand, produce each of the two hexanuclear units, which are symmetry related and of different quirality (see 1 and 2) (Fig. S10†). The 7^th^ (HV)^−^ ligand, which acts as *trans*-bichelate, bridges Cu7 and Cu8 to form the dimeric group (Fig. 4). The two hexamers are linked *via* one μ_2_-perchlorato ligand (Cl2) [bonds Cu1–O21(Cl2)O21a–Cu1a], with the O1⋯O1a distance being 6.816(9) Å, far longer than the equivalent distance in 1 and 2, as expected since in 3 the two hexanuclear units are not properly paired but rather shifted towards each other. One μ_4_-perchlorato ligand (Cl1) which coordinates on apical positions simultaneously Cu1, Cu2 (semi-coordination, 2.852(19) Å, in this case), Cu3 and Cu8 links one hexamer with the dinuclear moiety (Fig. 3a; see also S10†). There is one additional bridge, a second μ_2_-perchlorato ligand (Cl5) which connects Cu7 with Cu5 [Cu5–O51(Cl5)O52–Cu7]. 4 monodentate perchlorate anions coordinate axially to Cu4 (Cl3, Cl4), Cu5 (Cl6) and Cu6 (Cl7) (Fig. 3c and d). The other 2 perchlorate anions (Cl8 and Cl9) are not coordinating.

The chain grows through (a) another μ_4_-perchlorato ligand (Cl1a) which coordinates the Cu_3_-triangular copper centers of the second hexamer (Cu1a, Cu2a, Cu3a), with the Cu8a copper atom of another dimeric unit (Cu8a, Cu7a), and (b) a new μ_2_-perchlorato ligand (Cl5a) which links Cu5a and Cu7a (Fig. 3b).

In the hexanuclear unit the three ligand planes are visibly deviated from the plane defined by Cu1, Cu2 and Cu3. The large distortion is produced by the polydentate character of the coordinating perchlorate anions. Deviation of the central μ_3_-O1(H) atom toward the triangular Cu_3_ plane is 0.498(10)Å (slightly lower than in 1 and 2).

As for the coordination geometry of the copper sites, the 6Cu(ii) of the hexanuclear arrays display analogous distorted octahedral N_2_O_2_ + O_2_ environment but with different ligands on apical positions (Fig. 3b). On axial positions Cu1 presents two polydentate perchlorato ligands; Cu2 and Cu3 one (the) tetradentate perchlorate anion and one water molecule; Cu4 and Cu5, two perchlorate anions; Cu6 one perchlorate and one water molecule. The 2Cu(ii) centers of the dimeric moiety are NO_3_ + O penta-coordinated (Fig. 4). The basal positions of Cu7 are occupy by the N(triazole) and O(carbonyl) atoms of the ligand, and two O water atoms; the apical ones, by one O perchlorate atom (O52). To end, Cu8 is equatorially coordinated to the N(acetamido) and N(pyridine) atoms of the ligand and to two O water atoms, and apically to the O14 atom of the Cl1 perchlorate. Bond distances in the coordination polyhedron are listed in Table 2.

Finally to be indicated that in the dimeric unit the Cu7⋯Cu8 distance is 5.272(9) Å [the average Cu(central)–Cu(bridged-external) distance is 5.360(2) Å in the hexanuclear units of 3]. The Cu8⋯Cu8a distance in two consecutive Cu14 units of the chain is 20.387(9) Å whereas the Cu7⋯Cu7a distance is 22.117(9) Å.

**Fig. 5:**
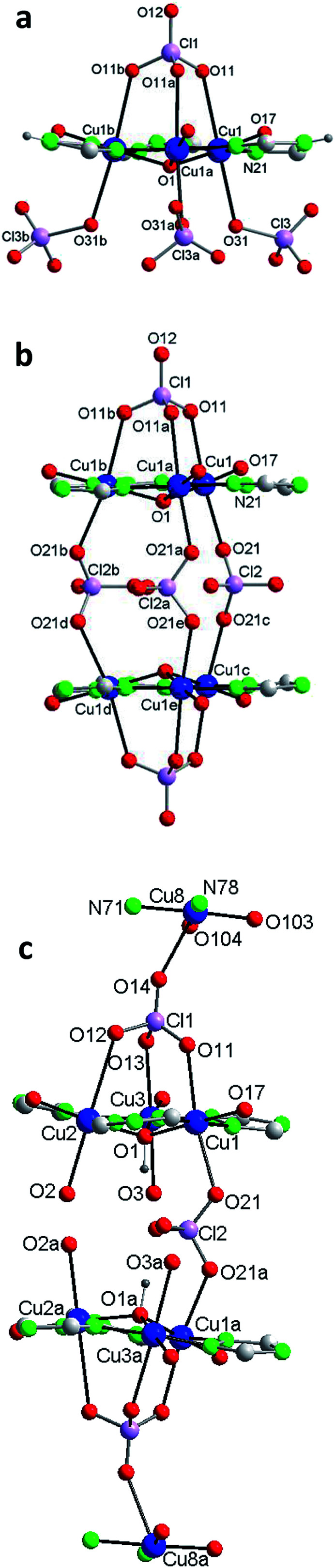
Detail of the trigonal clusters of Cu atoms in 1 (a), 2 (b) and 3 (c).

Fig. 5 shows the Cu_3_O cores of 1, 2 and 3 with the corresponding coordinating perchlorates (the Cu peripheral centers are not included) to emphasize the different association mediated by the perchlorate anions.

### Magnetic studies

**Fig. 6:**
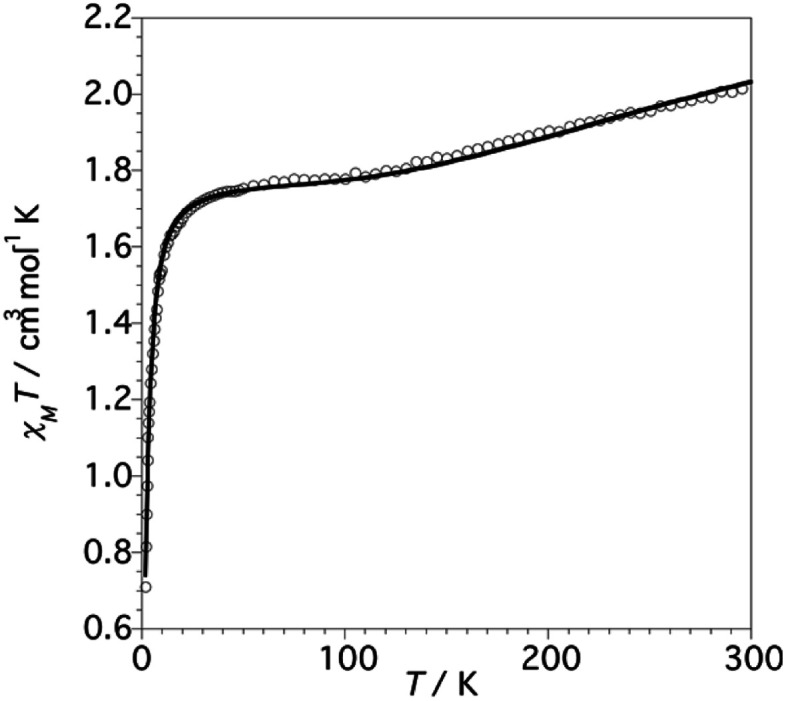
*χ*_M_ *T vs. T* plot for complex 1 (values per Cu_6_ unit). The solid line corresponds to the best-fit parameters obtained from the Hamiltonian (eqn 1).

The thermal dependence of the *χ*_M_*T* product for 1 (*χ*_M_ being the magnetic susceptibility per Cu_6_ unit) is shown in Fig. 6.

At 300 K the *χ*_M_*T* value is 2.05 cm^3^ K mol^−1^, which is lower than the value expected for six non-interacting Cu^II^*S* = 1/2 ions (∼2.4 cm^3^ K mol^−1^) with a reasonable *g*-value.^2,33^ Upon cooling, the *χ*_M_*T* product decreases slowly reaching a plateau at 1.75 cm^3^ K mol^−1^ between 90 and 60 K. Below *ca.* 20 K, *χ*_M_*T* decreases sharply to achieve a value of 0.70 cm^3^ K mol^−1^ at 2 K.

**Scheme 3:**
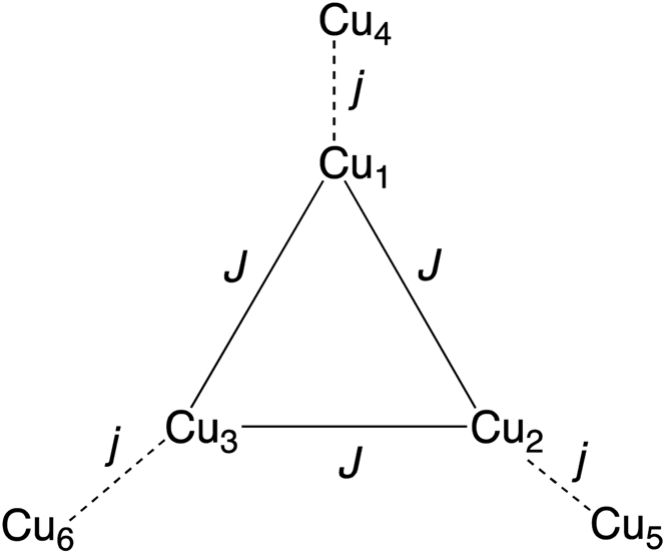
The two different antiferromagnetic exchange pathways in the Cu_6_ units of 1.

From structural analysis (Schemes 2 and 3) this behavior can be rationalized considering two steps. Firstly, the high-temperature region (300–100 K) reveals the presence of a moderate-to-strong antiferromagnetic interaction within the “triangular unit” between the Cu1, Cu2 and Cu3 centers, doubly bridged *via* the central oxo ligand and the peripheral N–N triazole atoms. This {Cu_3_(μ_3_-O/OH)(N–N)_3_} bridging system has already been described as leading to significant antiferromagnetic coupling.^7–9,33^ In previous studies we reported a series of trinuclear compounds of this type built with triazole ligands which exhibited *χ*_M_*T* values of 0.37–0.40 cm^3^ K mol^−1^ in the range 90–60 K.^7,8^ The plateau value of 1.75 cm^3^ K mol^−1^ for 1 would roughly correspond to the presence of the Cu_3_ triangle plus three almost isolated Cu^II^ additional centers (0.40 + 1.30).

Secondly, at lower temperatures (60–2 K), a weaker antiferromagnetic exchange involving the pairs Cu1–Cu4, Cu2–Cu5 and Cu3–Cu6 occurs. This weak antiferromagnetic interaction takes place through the double “NCN + NCO” bridge (Scheme 2), in a *trans*-bridging mode (following literature terminology),^34^ as observed previously in the “2 + 1 + 1 + 1 + 1” hexanuclear compound [Cu_6_(HdiV)_2_(ClO_4_)_6_(H_2_O)_14_](ClO_4_)_2_·10H_2_O (A).^32^

Considering all of these features and the symmetry of the structure (Cu_3_ is a perfect triangle) the magnetic data were simulated by means of the following C_3_-symmetric Hamiltonian (eqn 1):^2^1
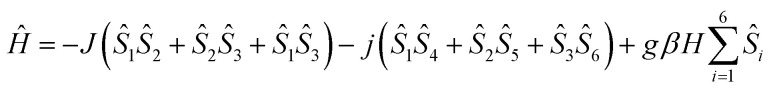


Fitting of the magnetic properties of Cu^II^ triangular clusters, example of spin frustrate systems, requires the use of both isotropic and ASE Hamiltonian terms to account for the low temperatures behavior.^7–9,33^ For 1, however, the experimental data were well reproduced without incorporation of the ASE term into the magneto-chemical analysis. Presumably the ASE is shadowed by the dominant antiferromagnetic interaction within the 3 dimeric Cu(ii) units. The resulting best-fit parameters were:*g* = 2.18(1), *J* = −247.0(1) cm^−1^ and *j* = −5.15(2) cm^−1^.

The high symmetry of the structure of 1 must be responsible of the high value of *J* (−247 cm^−1^), constant of the AF exchange within the tri-copper triangle, when compared with the values exhibited by the reported analogous triazole trimers (177–195 cm^−1^).^8^ In contrast, the *j* constant (−5.15 cm^−1^), corresponding to the AF exchange between the coupled “internal Cu-peripheral Cu” pairs is significantly lower than the one observed for a similar pathway in the hexanuclear compound A (−35 cm^−1^).^32^ The polydentate character of the perchlorate ligands on axial positions, which induces significant distortion on the equatorial planes of Cu1 (central) and Cu(2) (peripheral), could account for the lower value of *j*.^35^

EPR spectra at low *T* have been recorded in search of the ASE signature. The X-band powder spectrum of 1 at 4 K (Fig. S11†) displays signals at *g*_‖_ = 2.30 and *g*_⊥_ = 1.61, the last one being indeed indicative of the existence of ASE (*g* < 2.0).^8,9^

## Conclusions

We have prepared and tested a new simple 1,2,4-triazole ligand, H_2_V, capable of yielding with Cu(ii) a new building block consisting of hexanuclear Cu(ii) clusters with Cu_3_–O(H) core, that is, of the triangular type, but with expanded nuclearity: Cu_3_O(H)–3Cu. The hexanuclear units can aggregate in different forms. In the presence of perchlorate anions we have isolated and described three different structures with 6Cu (compound 1), 12Cu (compound 2) and 14Cu (compound 3) of nuclearity. The magnetic properties of the hexanuclear cluster have been studied with 1 and rationalized. Compound 2 combines high nuclearity and a porous 3D structure. Compound 3 is a mixed system (hexanuclear units and dimeric units) in which the dinuclear units link dodecanuclear assembles to afford a one-dimensional coordination polymer. While assembles of triangular Cu_3_O groups have been extensively described the introduction of the Cu_3_O–Cu_3_ fragment offers a straightforward way to enhance the magnetism of the resulting frameworks/CPs. We plan to explore the possibilities of this hexanuclear Cu(ii) scaffold with different anions and anionic linkers.

## Conflicts of interest

There are no conflicts to declare.

## Supplementary Material

RA-009-C9RA05922A-s001

RA-009-C9RA05922A-s002
