# Supplementary material for: Hexanuclear Cu3O–3Cu triazole-based units as novel core motifs for high nuclearity copper(ii) frameworks
Source: RSC Adv. 2019 Sep 17;9(50):29357–67. doi: 10.1039/c9ra05922a (PMC9071962; doi:10.1039/c9ra05922a)
Supplement: RA-009-C9RA05922A-s001 [file RA-009-C9RA05922A-s001.pdf]

Electronic Supplementary Material (ESI) for RSC Advances.
This journal is © The Royal Society of Chemistry 2019

Supplementary material (S1-S11) (17 pages)

Hexanuclear Cu_3O−3Cu Triazole-Based Units as Novel Core Motifs for High Nuclearity Copper(II) Frameworks

S. Ferrer,[§,*] J. Hernández-Gil,[§] F.J. Valverde-Muñoz,[#] F. Lloret,[#] and A. Castiñeiras[‡]

[§]Departament de Química Inorgànica, Universitat de València, Spain.

[#]Institut de Ciència Molecular, Universitat de València, Spain.

[‡]Departamento de Química Inorgánica, Universidad de Santiago de Compostela, Santiago de Compostela, Spain.

S1 Crystal Structure Determination of compound 1:

A green cubic-like crystal of $[Cu_6(HV)_3(ClO_4)_7(O)(H_2O)_9]\cdot 8H_2O$ was mounted on a glass fiber and used for data collection. Crystal data were collected at 100(2) K, using a Bruker SMART CCD 1000 diffractometer. Graphite monochromated MoKα radiation (λ = 0.71073 Å) was used throughout. The data were processed with SAINT [1] and corrected for absorption using SADABS (transmissions factors: 1.000 - 0.764) [2]. The structure was solved by direct methods using the program SHELXS-2013 [3] and refined by full-matrix least-squares techniques againts F^2 using SHELXL-2013 [3]. Positional and anisotropic atomic displacement parameters were refined for all nonhydrogen atoms. Solvent molecules (observed from the difference Fourier maps) could not be identified because of significant disorder and/or partial occupancy, they were SQUEEZE'd out using PLATON [5]. However, this has not been taken into account in the density value and the formula reported in the CIF file and tables. Final refinement (SHELXL-2013) included a) the displacement-coefficient restrains ISOR of 0.01 for CL3, O31, O32, O33, O34, O7, and O8 to prevent these atoms can be split into two positions, and b) bond-length restrains DFIX of 1.50(5) angstrom's for CL3-O31, CL3-O32, CL3-O33, CL3-O34. The problem of disorder around the perchlorate site gave rise to rather elongated ellipsoids for Cl3, O31, O32, O33 and O34. Hydrogen atoms were located in difference maps and included as fixed contributions riding on attached atoms with isotropic thermal parameters 1.2 times those of their carrier atoms. The H atoms of crystallization water molecules, O(5), O(6), O(7) and O(8) were not located. The large discrepancy between the *R* and *wR* values is due to the high thermal vibration present in perchlorate groups and crystallization water molecules. The low accuracy of the structure refinement derives from the weak diffracting power of the crystals. Nevertheless, the final results can be considered satisfactory from the chemical point of view. Criteria of a satisfactory complete analysis were the ratios of "rms" shift to standard deviation less than 0.001. The lowest (-1.35 $e\cdot Å^{-3}$) and highest (1.94 $e\cdot Å^{-3}$) peaks in the final difference Fourrier map are located close to the Cl(3) and O32 atoms at distances of 0.63 and 1.69 Å, respectively. Atomic scattering factors from "International Tables for Crystallography" [4]. Molecular graphics from PLATON [5] and DIAMOND [6]. A summary of the crystal data, experimental details and refinement results are listed in table 1.

References
[1] Bruker (1997). SMART and SAINT. Area Detector Control and Integration Software, Bruker Analytical X-ray Instruments Inc., Madison, Wisconsin, USA.
[2] G.M. Sheldrick (2001). SADABS. Program for Empirical Absorption Correction of Area Detector Data. University of Goettingen, Germany.
[3] G.M. Sheldrick (2015). Crystal structure refinement with SHELXL. Acta Crystallogr. Sect. C. 71, 3-8.
[4] A.J.C. Wilson (1995). International Tables for Crystallography. Vol. C, Kluwer Academic Publishers: Dordrecht, The Netherlands.
[5] A.L. Spek. (2009). Acta Cryst. D65, 148-155.
[6] H. Putz & K. Brandenburg (2018). DIAMOND Version 4.5.3, Crystal Impact GbR: Bonn, Germany.

S2 Crystal Structure Determination of compound 2:

A green hexagonal-prismatic crystal of $[Cu_{12}(O)_2(V)_6(ClO_4)_5(H_2O)_{18}](ClO_4)_3\cdot 6(H_2O)$ was mounted on a glass fiber and used for data collection. Crystal data were collected at 100(2) K, using a Bruker SMART CCD 1000 diffractometer. Graphite monochromated MoKα radiation (λ = 0.71073 Å) was used throughout. The data were processed with SAINT [1] and corrected for absorption using SADABS (transmissions factors: 1.000 - 0.756) [2]. The structure was solved by direct methods using the program SHELXS-2013 [3] and refined by full-matrix least-squares techniques againts F^2 using SHELXL-2013 [3]. Positional and anisotropic atomic displacement parameters were refined for all nonhydrogen atoms. The $Cu(H_2O)_3$ fragment of the metal coordination sphere, Cu2, O2, O3, O4, was assumed to occupy two alternative orientations inclined at an angle of 35(1)° given an occupancy of 0.5 for each position (Cu2A/Cu2B, O2A/O2B, O3A/O3B, O4A/O4B). Solvent molecules (observed from the difference Fourier maps) could not be identified because of significant disorder and/or partial occupancy, they were SQUEEZE'd out using PLATON [5]. Final refinement (SHELXL-2013) included the SADI instruction for perchlorate ions of Cl1, Cl2, and Cl3, to restrain these ions to be a regular tetrahedron. Hydrogen atoms attached to carbon and nitrogen atoms were placed in geometrically idealized positions and refined using a riding model. Hydrogen atoms on water molecules were not located. In the final formula are included the hydrogen atoms nonlocalized. The large discrepancy between the *R* and *wR* values is due to the splitting of the $Cu(H_2O)_3$ fragment, the high thermal vibration present in perchlorate groups, and crystallization water molecules. The low accuracy of the structure refinement derives from the weak diffracting power of the crystals. Nevertheless, the final results can be considered satisfactory from the chemical point of view. Criteria of a satisfactory complete analysis were the ratios of "rms" shift to standard deviation less than 0.001. The lowest (-0.50 e$\cdot$Å^{-3}) and highest (1.41 e$\cdot$Å^{-3}) peaks in the final difference Fourrier map are located close to the Cl(2) and O(12) atoms at distances of 1.12 and 1.46 Å, respectively. Atomic scattering factors from "International Tables for Crystallography" [4]. Molecular graphics from PLATON [5] and DIAMOND [6]. A summary of the crystal data, experimental details and refinement results are listed in table 1.

References
[1] Bruker (1997). SMART and SAINT. Area Detector Control and Integration Software, Bruker Analytical X-ray Instruments Inc., Madison, Wisconsin, USA.
[2] G.M. Sheldrick (2001). SADABS. Program for Empirical Absorption Correction of Area Detector Data. University of Goettingen, Germany.
[3] G.M. Sheldrick (2015). Crystal structure refinement with SHELXL. Acta Crystallogr. Sect. C. 71, 3-8.
[4] A.J.C. Wilson (1995). International Tables for Crystallography. Vol. C, Kluwer Academic Publishers: Dordrecht, The Netherlands.
[5] A.L. Spek. (2009). Acta Cryst. D65, 148-155.
[6] H. Putz & K. Brandenburg (2018). DIAMOND Version 4.5.3, Crystal Impact GbR: Bonn, Germany.

S3 Crystal Structure Determination of compound 3:

A green long-prismatic crystal of $\{[Cu_{14}(OH)_2(V)_6(HV)(ClO_4)_{11}(H_2O)_{20}](ClO_4)_2\cdot 14H_2O\}_n$ was mounted on a glass fiber and used for data collection. Crystal data were collected at 100(2) K, using a Bruker X8 KappaAPEXII diffractometer. Graphite monochromated MoKα radiation (λ = 0.71073 angstrom's) was used throughout. The data were processed with APEX2 [1] and corrected for absorption using SADABS (transmissions factors: 1.000 - 0.893) [2]. The structure was solved by direct methods using the program SHELXS-2013 [3] and refined by full-matrix least-squares techniques againts F^2 using SHELXL-2013 [3]. Positional and anisotropic atomic displacement parameters were refined for Cu, Cl, and the coordinated nitrogen and oxygen atoms, and isotropically for the remaining nonhydrogen atoms. Solvent molecules (observed from the difference Fourier maps) could not be identified because of significant disorder and/or partial occupancy, they were SQUEEZE'd out using PLATON [5]. Final refinement (SHELXL-2013) included a) the displacement-coefficient restrains ISOR of 0.005 for O77, N78, N80, O10, O12, O13, and O14 to prevent these atoms can be split into two positions or 'non-positive definite', and SADI for perchlorate ions of Cl1, Cl3, Cl6, Cl7 and Cl8, to restrain these ions to be a regular tetrahedron, and b) instructions AFIX 66 and AFIX 59 to perform rigid group refinements for pyridyl ring, N71/C72/C73/C74/C75/C76, and triazolyl ring, C79/N80/N81/C82/N83, respectively. Special position constraints for Cl2 and Cl9. Hydrogen atoms attached to carbon and nitrogen atoms were placed in geometrically idealized positions and refined using a riding model. Hydrogen atoms on water molecules were not located. In the final formula are included the hydrogen atoms nonlocalized. The large discrepancy between the R and wR values is due to the high thermal vibration present in perchlorate groups and crystallization water molecules. The low accuracy of the structure refinement derives from the weak diffracting power of the crystals. Nevertheless, the final results can be considered satisfactory from the chemical point of view. Criteria of a satisfactory complete analysis were the ratios of "rms" shift to standard deviation less than 0.001. The lowest (-1.28 $e\cdot Å^{-3}$) and highest (3.27 $e\cdot Å^{-3}$) peaks in the final difference Fourrier map are located close to the O(72) and H(82A) atoms at distances of 0.83 and 1.45 Å, respectively. Atomic scattering factors from "International Tables for Crystallography" [4]. Molecular graphics from PLATON [5] and DIAMOND [6]. A summary of the crystal data, experimental details and refinement results are listed in table 1.

References
[1] Bruker (1997). SMART and SAINT. Area Detector Control and Integration Software, Bruker Analytical X-ray Instruments Inc., Madison, Wisconsin, USA. [2] G.M. Sheldrick (2001). SADABS. Program for Empirical Absorption Correction of Area Detector Data. University of Goettingen, Germany. [3] G.M. Sheldrick (2015). Sheldrick, G. M. Crystal structure refinement with SHELXL. Acta Crystallogr. Sect. C. 2015, 71, 3-8.[4] A. J. C. Wilson (1995). International Tables for Crystallography. Vol. C, Kluwer Academic Publishers: Dordrecht, The Netherlands. [5] A.L. Spek. (2009). Acta Cryst. D65, 148-155. [6] H. Putz & K. Brandenburg (2018). DIAMOND Version 4.5.3, Crystal Impact GbR: Bonn, Germany.

S4 Table. Selected bond lengths [Å] and angles [°] for $[Cu_6(HV)_3(ClO_4)_7(O)(H_2O)_9]\cdot 8H_2O$ (**1**)

Cu(1)-N(20)	1.909(6)
Cu(1)-N(21)	1.957(6)
Cu(1)-O(17)	1.969(6)
Cu(1)-O(1)	1.995(3)
Cu(1)-O(31)	2.501(11)
Cu(1)-O(11)	2.634(14)
Cu(1)-Cu(1)#1	3.3437(16)
Cu(1)-Cu(1)#2	3.3437(16)
Cu(1)-Cu(2)	5.3448(16)
Cu(2)-O(3)	1.972(8)
Cu(2)-N(11)	1.968(8)
Cu(2)-O(2)	1.994(8)
Cu(2)-N(18)	2.008(6)
Cu(2)-O(21)	2.470(11)
Cu(2)-O(4)	2.69(3)
N(20)-Cu(1)-N(21)	177.5(4)
N(20)-Cu(1)-O(17)	86.5(2)
N(21)-Cu(1)-O(17)	93.7(2)
N(20)-Cu(1)-O(1)	89.70(19)
N(21)-Cu(1)-O(1)	90.04(19)
O(17)-Cu(1)-O(1)	175.5(3)
N(20)-Cu(1)-O(31)	92.0(4)
N(21)-Cu(1)-O(31)	90.5(4)
O(17)-Cu(1)-O(31)	86.2(4)
O(1)-Cu(1)-O(31)	96.3(5)
N(20)-Cu(1)-O(11)	84.3(4)
N(21)-Cu(1)-O(11)	93.3(4)
O(17)-Cu(1)-O(11)	86.6(3)
O(1)-Cu(1)-O(11)	90.6(4)
O(31)-Cu(1)-O(11)	172.1(4)
N(20)-Cu(1)-Cu(1)#1	118.66(18)
N(21)-Cu(1)-Cu(1)#1	60.41(18)
O(17)-Cu(1)-Cu(1)#1	149.00(18)
O(1)-Cu(1)-Cu(1)#1	33.08(14)
O(31)-Cu(1)-Cu(1)#1	109.0(3)
O(11)-Cu(1)-Cu(1)#1	78.8(2)
N(20)-Cu(1)-Cu(1)#2	58.80(18)
N(21)-Cu(1)-Cu(1)#2	120.39(18)
O(17)-Cu(1)-Cu(1)#2	142.49(18)
O(1)-Cu(1)-Cu(1)#2	33.08(14)
O(31)-Cu(1)-Cu(1)#2	107.0(3)
O(11)-Cu(1)-Cu(1)#2	76.9(2)
Cu(1)#1-Cu(1)-Cu(1)#2	60.0
N(20)-Cu(1)-Cu(2)	47.16(18)
N(21)-Cu(1)-Cu(2)	133.42(19)

O(17)-Cu(1)-Cu(2)	40.54(16)
O(1)-Cu(1)-Cu(2)	136.15(5)
O(31)-Cu(1)-Cu(2)	80.4(3)
O(11)-Cu(1)-Cu(2)	92.0(3)
Cu(1)#1-Cu(1)-Cu(2)	164.44(3)
Cu(1)#2-Cu(1)-Cu(2)	105.88(4)
O(3)-Cu(2)-N(11)	92.2(3)
O(3)-Cu(2)-O(2)	89.6(3)
N(11)-Cu(2)-O(2)	176.0(5)
O(3)-Cu(2)-N(18)	170.0(5)
N(11)-Cu(2)-N(18)	82.5(3)
O(2)-Cu(2)-N(18)	96.3(3)
O(3)-Cu(2)-O(21)	86.5(5)
N(11)-Cu(2)-O(21)	97.3(4)
O(2)-Cu(2)-O(21)	86.4(4)
N(18)-Cu(2)-O(21)	85.9(3)
O(3)-Cu(2)-O(4)	95.9(6)
N(11)-Cu(2)-O(4)	87.3(5)
O(2)-Cu(2)-O(4)	88.9(5)
N(18)-Cu(2)-O(4)	92.3(5)
O(21)-Cu(2)-O(4)	174.7(4)
O(3)-Cu(2)-Cu(1)	169.1(4)
N(11)-Cu(2)-Cu(1)	78.4(2)
O(2)-Cu(2)-Cu(1)	100.2(2)
N(18)-Cu(2)-Cu(1)	5.1(2)
O(21)-Cu(2)-Cu(1)	89.5(2)
O(4)-Cu(2)-Cu(1)	89.0(5)
Cu(1)#2-O(1)-Cu(1)#1	113.8(3)
Cu(1)#2-O(1)-Cu(1)	113.8(3)
Cu(1)#1-O(1)-Cu(1)	113.8(3)

Symmetry transformations used to generate equivalent atoms:
#1 -y+1,x-y+1,z   #2 -x+y,-x+1,z

S5 Table. Selected bond lengths [Å] and angles [°] for $[Cu_{12}(O)_2(V)_6(ClO_4)_5(H_2O)_{18}](ClO_4)_3\cdot 6(H_2O)$ (**2**).

Cu(1)-N(20)	1.903(6)
Cu(1)-N(21)	1.947(6)
Cu(1)-O(17)	1.964(5)
Cu(1)-O(1)	2.009(3)
Cu(1)-O(21)	2.417(7)
Cu(1)-O(11)	2.506(8)
Cu(1)-Cu(1)#1	3.3616(16)
Cu(1)-Cu(1)#2	3.3616(16)
Cu(1)-Cu(2A)	5.256(3)
Cu(1)-Cu(2B)	5.365(3)
Cu(1)-Cu(1)#3	6.687(2)
Cu(1)-Cu(1)#4	7.485(2)
Cu(2A)-O(3A)	1.933(18)
Cu(2A)-O(2A)	2.005(18)
Cu(2A)-N(11)	2.009(9)
Cu(2A)-N(18)	2.011(8)
Cu(2A)-O(4A)	2.21(4)
Cu(2B)-N(11)	1.971(8)
Cu(2B)-O(2B)	2.005(19)
Cu(2B)-N(18)	2.010(8)
Cu(2B)-O(3B)	2.014(16)
Cu(2B)-O(4B)	2.39(6)
O(1)-Cu(1)#1	2.009(3)
O(1)-Cu(1)#2	2.009(3)
O(1)-O(1)#4	5.648(18)
N(20)-Cu(1)-N(21)	178.7(3)
N(20)-Cu(1)-O(17)	86.7(2)
N(21)-Cu(1)-O(17)	94.5(2)
N(20)-Cu(1)-O(1)	88.98(18)
N(21)-Cu(1)-O(1)	89.80(17)
O(17)-Cu(1)-O(1)	175.61(16)
N(20)-Cu(1)-O(21)	88.9(3)
N(21)-Cu(1)-O(21)	91.8(3)
O(17)-Cu(1)-O(21)	80.2(3)
O(1)-Cu(1)-O(21)	99.0(3)
N(20)-Cu(1)-O(11)	89.6(3)
N(21)-Cu(1)-O(11)	89.9(3)
O(17)-Cu(1)-O(11)	90.4(2)
O(1)-Cu(1)-O(11)	90.2(3)
O(21)-Cu(1)-O(11)	170.6(3)
O(3A)-Cu(2A)-O(2A)	92.1(9)
O(3A)-Cu(2A)-N(11)	169.4(8)
O(2A)-Cu(2A)-N(11)	95.8(8)
O(3A)-Cu(2A)-N(18)	93.0(5)
O(2A)-Cu(2A)-N(18)	172.5(11)

N(11)-Cu(2A)-N(18)	79.9(3)
O(3A)-Cu(2A)-O(4A)	86(2)
O(2A)-Cu(2A)-O(4A)	85.7(14)
N(11)-Cu(2A)-O(4A)	102(2)
N(18)-Cu(2A)-O(4A)	89.2(10)
N(11)-Cu(2B)-O(2B)	93.5(5)
N(11)-Cu(2B)-N(18)	80.8(3)
O(2B)-Cu(2B)-N(18)	172.3(8)
N(11)-Cu(2B)-O(3B)	168.5(11)
O(2B)-Cu(2B)-O(3B)	89.3(7)
N(18)-Cu(2B)-O(3B)	97.3(6)
N(11)-Cu(2B)-O(4B)	112.3(17)
O(2B)-Cu(2B)-O(4B)	94.4(14)
N(18)-Cu(2B)-O(4B)	92.7(11)
O(3B)-Cu(2B)-O(4B)	56.3(18)
Cu(1)#1-O(1)-Cu(1)	113.6(2)
Cu(1)#1-O(1)-Cu(1)#2	113.6(2)
Cu(1)-O(1)-Cu(1)#2	113.6(2)

Symmetry transformations used to generate equivalent atoms:
#1 -y+1,x-y,z   #2 -x+y+1,-x+1,z   #3 x,y,-z+1/2
#4 -x+y+1,-x+1,-z+1/2

S6 Table. Selected bond lengths [Å] and angles [°] for $\{[Cu_{14}(OH)_2(V)_6(HV)(ClO_4)_{11}(H_2O)_{20}](ClO_4)_2\cdot14H_2O\}_n$ (**3**)

Cu(1)-N(20)	1.912(10)
Cu(1)-N(41)	1.945(10)
Cu(1)-O(1)	1.986(8)
Cu(1)-O(17)	1.968(9)
Cu(1)-O(21)	2.403(13)
Cu(1)-O(11)	2.450(12)
Cu(1)-Cu(3)	3.335(2)
Cu(1)-Cu(2)	3.353(2)
Cu(1)-Cu(5)	5.361(2)
Cu(2)-N(60)	1.910(10)
Cu(2)-N(21)	1.935(10)
Cu(2)-O(57)	1.968(9)
Cu(2)-O(1)	1.994(9)
Cu(2)-O(2)	2.385(11)
Cu(2)-O(12)	2.852(19)
Cu(2)-Cu(3)	3.362(2)
Cu(2)-Cu(6)	5.367(2)
Cu(3)-N(40)	1.895(10)
Cu(3)-N(61)	1.955(11)
Cu(3)-O(37)	1.972(9)
Cu(3)-O(1)	2.012(8)
Cu(3)-O(3)	2.387(17)
Cu(3)-O(13)	2.546(15)
Cu(3)-Cu(4)	5.352(3)
Cu(4)-N(38)	2.009(11)
Cu(4)-N(31)	1.974(12)
Cu(4)-O(5)	1.988(12)
Cu(4)-O(4)	1.989(12)
Cu(4)-O(31)	2.265(18)
Cu(4)-O(41)	2.469(13)
Cu(5)-N(11)	1.965(12)
Cu(5)-O(6)	1.983(11)
Cu(5)-N(18)	2.003(10)
Cu(5)-O(7)	2.003(11)
Cu(5)-O(61)	2.422(15)
Cu(5)-O(51)	2.456(17)
Cu(6)-N(51)	1.960(12)
Cu(6)-O(9)	1.995(11)
Cu(6)-N(58)	2.007(12)
Cu(6)-O(10)	1.994(15)
Cu(6)-O(8)	2.258(19)
Cu(6)-O(71)	2.423(14)
Cu(7)-O(77)	1.76(3)
Cu(7)-N(80)	1.85(3)
Cu(7)-O(101)	1.94(5)
Cu(7)-O(102)	2.11(6)

Cu(7)-O(52)	2.67(2)
Cu(7)-Cu(8)	5.272(9)
Cu(8)-O(103)	1.88(3)
Cu(8)-N(71)	1.964(16)
Cu(8)-N(78)	1.95(3)
Cu(8)-O(104)	2.00(3)
Cu(8)-O(14)	2.498(18)
N(20)-Cu(1)-N(41)	178.8(4)
N(20)-Cu(1)-O(1)	88.9(4)
N(41)-Cu(1)-O(1)	90.0(4)
N(20)-Cu(1)-O(17)	87.2(4)
N(41)-Cu(1)-O(17)	93.9(4)
O(1)-Cu(1)-O(17)	175.6(4)
N(20)-Cu(1)-O(21)	91.0(5)
N(41)-Cu(1)-O(21)	89.7(5)
O(1)-Cu(1)-O(21)	97.5(4)
O(17)-Cu(1)-O(21)	80.5(5)
N(20)-Cu(1)-O(11)	89.4(5)
N(41)-Cu(1)-O(11)	90.2(5)
O(1)-Cu(1)-O(11)	92.7(4)
O(17)-Cu(1)-O(11)	89.4(4)
O(21)-Cu(1)-O(11)	169.9(4)
N(60)-Cu(2)-N(21)	172.8(5)
N(60)-Cu(2)-O(57)	86.5(4)
N(21)-Cu(2)-O(57)	93.6(4)
N(60)-Cu(2)-O(1)	90.0(4)
N(21)-Cu(2)-O(1)	89.3(4)
O(57)-Cu(2)-O(1)	174.1(4)
N(60)-Cu(2)-O(2)	95.8(5)
N(21)-Cu(2)-O(2)	91.4(5)
O(57)-Cu(2)-O(2)	93.0(4)
O(1)-Cu(2)-O(2)	92.0(4)
N(60)-Cu(2)-O(12)	88.1(5)
N(21)-Cu(2)-O(12)	84.7(5)
O(57)-Cu(2)-O(12)	86.4(4)
O(1)-Cu(2)-O(12)	88.8(4)
O(2)-Cu(2)-O(12)	176.0(5)
N(40)-Cu(3)-N(61)	176.2(5)
N(40)-Cu(3)-O(37)	86.4(4)
N(61)-Cu(3)-O(37)	94.3(4)
N(40)-Cu(3)-O(1)	89.2(4)
N(61)-Cu(3)-O(1)	90.0(4)
O(37)-Cu(3)-O(1)	175.1(4)
N(40)-Cu(3)-O(3)	91.1(6)
N(61)-Cu(3)-O(3)	92.6(6)
O(37)-Cu(3)-O(3)	93.5(6)
O(1)-Cu(3)-O(3)	88.8(6)
N(40)-Cu(3)-O(13)	90.2(5)
N(61)-Cu(3)-O(13)	86.1(5)
O(37)-Cu(3)-O(13)	89.8(5)

O(1)-Cu(3)-O(13)	88.1(4)
O(3)-Cu(3)-O(13)	176.6(6)
N(38)-Cu(4)-N(31)	82.2(5)
N(38)-Cu(4)-O(5)	169.6(6)
N(31)-Cu(4)-O(5)	92.7(5)
N(38)-Cu(4)-O(4)	96.3(4)
N(31)-Cu(4)-O(4)	175.2(6)
O(5)-Cu(4)-O(4)	89.5(5)
N(38)-Cu(4)-O(31)	100.3(7)
N(31)-Cu(4)-O(31)	90.4(7)
O(5)-Cu(4)-O(31)	88.7(7)
O(4)-Cu(4)-O(31)	85.4(7)
N(38)-Cu(4)-O(41)	86.4(5)
N(31)-Cu(4)-O(41)	96.6(5)
O(5)-Cu(4)-O(41)	85.2(5)
O(4)-Cu(4)-O(41)	87.8(5)
O(31)-Cu(4)-O(41)	170.9(6)
N(11)-Cu(5)-O(6)	92.2(5)
N(11)-Cu(5)-N(18)	82.2(4)
O(6)-Cu(5)-N(18)	170.1(6)
N(11)-Cu(5)-O(7)	175.2(6)
O(6)-Cu(5)-O(7)	89.3(5)
N(18)-Cu(5)-O(7)	97.0(4)
N(11)-Cu(5)-O(61)	92.7(5)
O(6)-Cu(5)-O(61)	92.3(6)
N(18)-Cu(5)-O(61)	96.0(5)
O(7)-Cu(5)-O(61)	82.7(6)
N(11)-Cu(5)-O(51)	97.9(5)
O(6)-Cu(5)-O(51)	85.9(6)
N(18)-Cu(5)-O(51)	86.9(5)
O(7)-Cu(5)-O(51)	86.8(6)
O(61)-Cu(5)-O(51)	169.3(5)
N(51)-Cu(6)-O(9)	178.9(5)
N(51)-Cu(6)-N(58)	81.8(5)
O(9)-Cu(6)-N(58)	97.1(5)
N(51)-Cu(6)-O(10)	90.8(6)
O(9)-Cu(6)-O(10)	90.2(5)
N(58)-Cu(6)-O(10)	168.9(6)
N(51)-Cu(6)-O(8)	92.8(6)
O(9)-Cu(6)-O(8)	87.7(6)
N(58)-Cu(6)-O(8)	94.8(7)
O(10)-Cu(6)-O(8)	93.8(8)
N(51)-Cu(6)-O(71)	97.6(5)
O(9)-Cu(6)-O(71)	82.0(5)
N(58)-Cu(6)-O(71)	86.8(5)
O(10)-Cu(6)-O(71)	86.0(6)
O(8)-Cu(6)-O(71)	169.7(5)
O(77)-Cu(7)-N(80)	92.1(15)
O(77)-Cu(7)-O(101)	89.4(18)
N(80)-Cu(7)-O(101)	170.0(18)
O(77)-Cu(7)-O(102)	178(2)

N(80)-Cu(7)-O(102)	90(2)
O(101)-Cu(7)-O(102)	89(2)
O(77)-Cu(7)-O(52)	97.1(13)
N(80)-Cu(7)-O(52)	88.5(11)
O(101)-Cu(7)-O(52)	81.5(15)
O(102)-Cu(7)-O(52)	83.9(18)
O(103)-Cu(8)-N(71)	177.9(11)
O(103)-Cu(8)-N(78)	97.2(13)
N(71)-Cu(8)-N(78)	84.0(11)
O(103)-Cu(8)-O(104)	83.5(11)
N(71)-Cu(8)-O(104)	95.2(10)
N(78)-Cu(8)-O(104)	174.8(12)
O(103)-Cu(8)-O(14)	113.1(10)
N(71)-Cu(8)-O(14)	65.3(8)
N(78)-Cu(8)-O(14)	80.8(11)
O(104)-Cu(8)-O(14)	94.1(9)

Symmetry transformations used to generate equivalent atoms:

S7 Figure which compares **1** and **2**

(a) Pairing of two hexanuclear units of **1** (two schematic views)

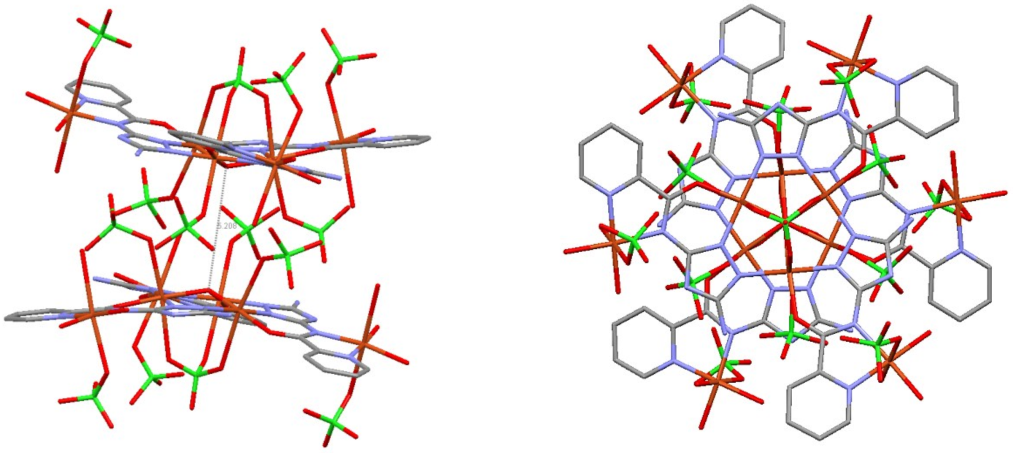

(b) The dodecanuclear unit of **2** (two schematic views)

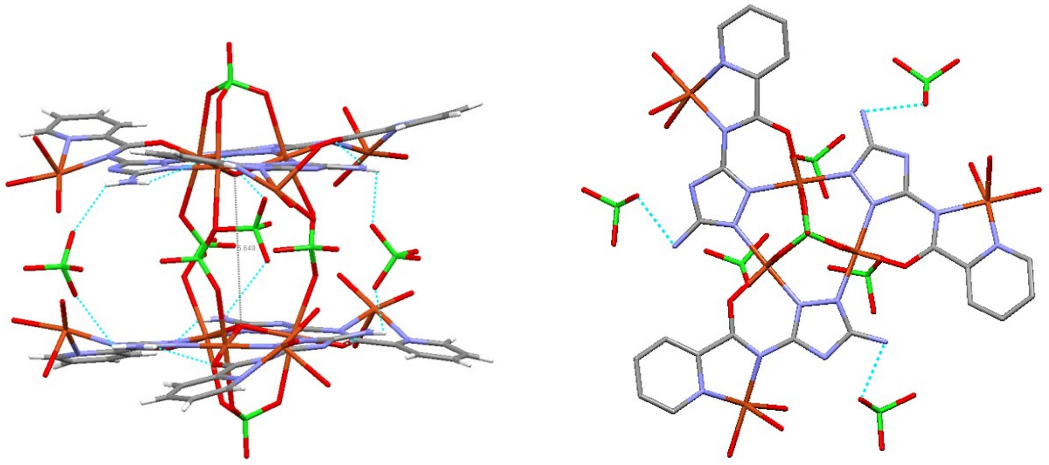

S8 Additional Figures for Compound **1**

(a) Schematic view of a chain *of pairs* of hexanuclear units of **1**.

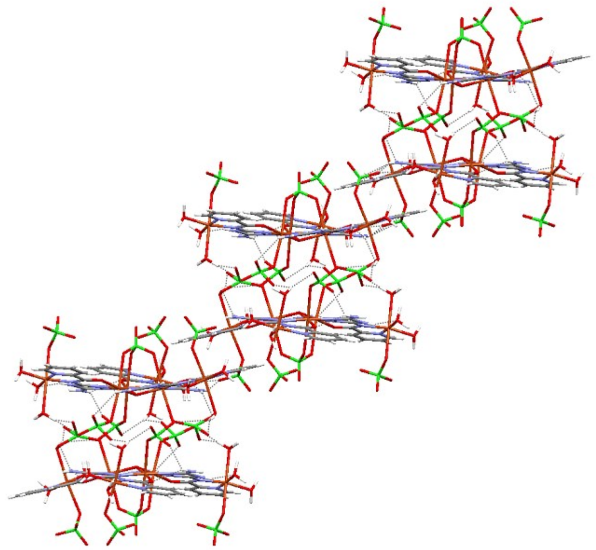

(b) Packing diagram of **1** viewed along the *c*-axis.

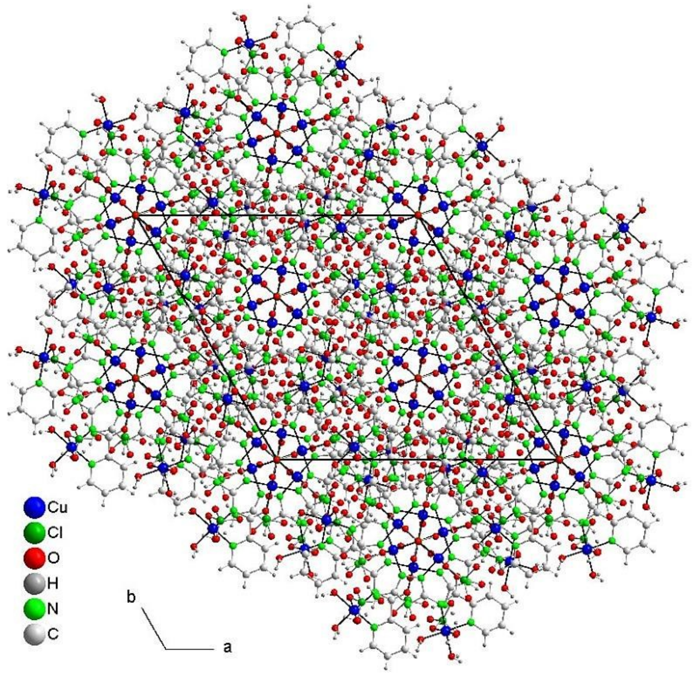

S9 Additional Figure for Compound **2**

Packing diagram of **2** projected on the plane *ab*. Hydrogen bonds are shown as orange dashed lines.

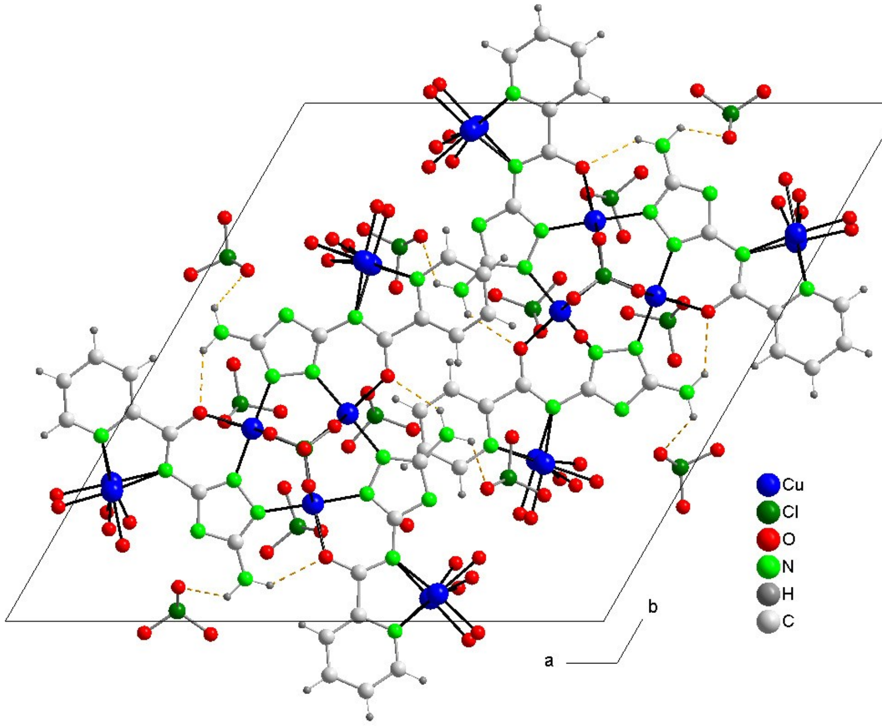

S10 Additional Figures for Compound **3**

(a) Perspective view of the hexanuclear fragment in the asymmetrical unit of **3** showing the coordination geometry of each copper atom. **(b)** Perspective of the molecular structure of **3** projected onto the *ab* plane.

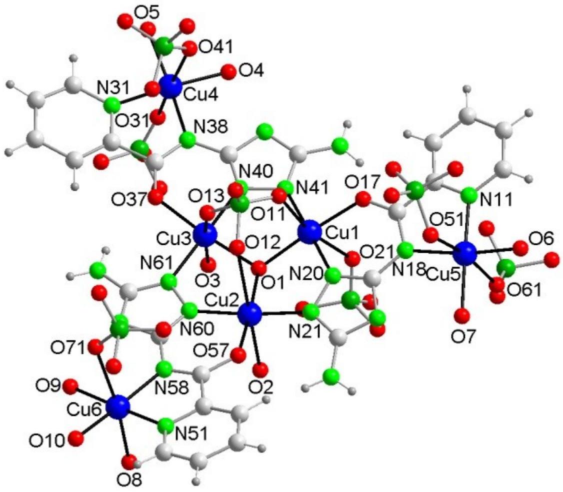

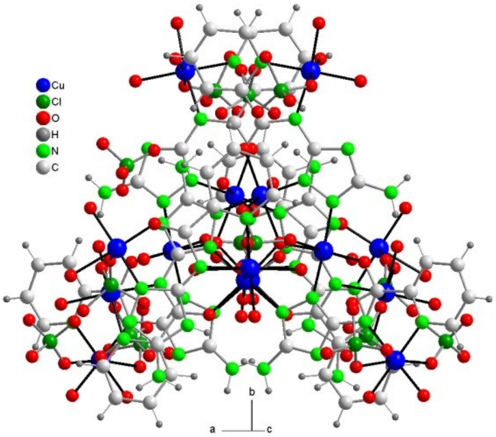

(c) Packing diagram of **3** projected onto the plane *ac*. **(d)** Schematic view of one of the tetradecanuclear "···Cu_2−(Cu_3O–3Cu)−(Cu_3O–3Cu)−Cu_2'···" copper(II) chains.

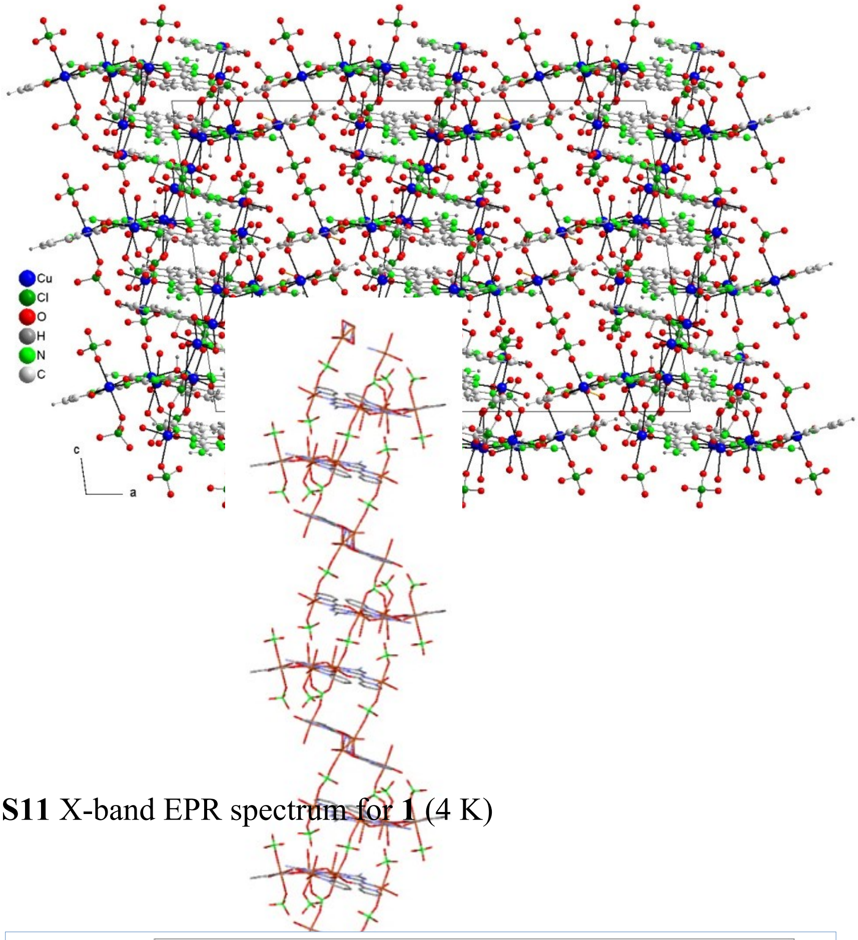

S11 X-band EPR spectrum for **1** (4 K)

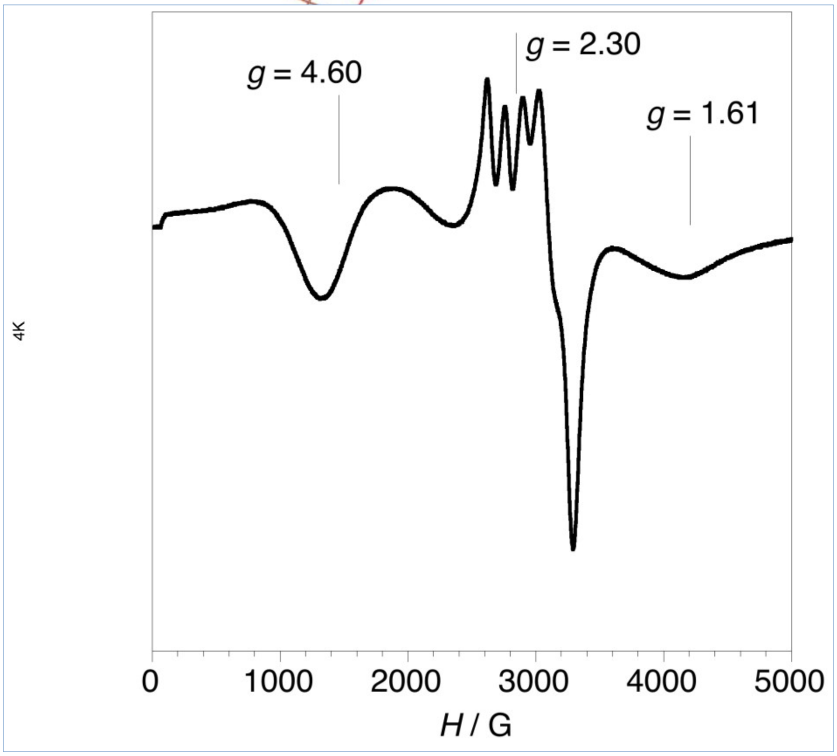

The X-band powder EPR spectrum of **1** has been registered at 4 K.

For the spin doublet ground state, S = 1/2, corresponding to a triangular system of three antisymmetrically coupled Cu(II) ions, two strong EPR transitions are expected at moderate magnetic fields, which origin the g parallel and g perpendicular resonances. For **1**, in the 2000-3100 region it is observed one multiplet centered at g = 2.30 which can be assigned to $g_{\|}$. This band is split in 4 peaks due to the coupling with the nuclear spin of Cu (I = 3/2), being the resultant hyperfine splitting constant of A = 136 G. Between 3100-5000 G the spectrum shows the perpendicular signal as a broad band at g = 1.61. This resonance at high fields (g < 2) is the signature for the existence of ASE within the central trinuclear Cu_3O group of the compound. Finally, there is a third feature, a broad band at half field (g = 4.60) which can be attributed to the occurrence of a spin triplet state, S = 1, close in energy to the spin singlet ground state, S = 0.
